# Emerging Multiplex Nucleic Acid Diagnostic Tests for Combating COVID-19

**DOI:** 10.3390/bios12110978

**Published:** 2022-11-07

**Authors:** Patarajarin Akarapipad, Elizabeth Bertelson, Alexander Pessell, Tza-Huei Wang, Kuangwen Hsieh

**Affiliations:** 1 Department of Biomedical Engineering, Johns Hopkins University, Baltimore, MD 21218, USA; 2 Department of Mechanical Engineering, Johns Hopkins University, Baltimore, MD 21218, USA

**Keywords:** multiplex, COVID-19, SARS-CoV-2, virus, nucleic acid amplification testing, point-of-care

## Abstract

The COVID-19 pandemic caused by SARS-CoV-2 has drawn attention to the need for fast and accurate diagnostic testing. Concerns from emerging SARS-CoV-2 variants and other circulating respiratory viral pathogens further underscore the importance of expanding diagnostic testing to multiplex detection, as single-plex diagnostic testing may fail to detect emerging variants and other viruses, while sequencing can be too slow and too expensive as a diagnostic tool. As a result, there have been significant advances in multiplex nucleic-acid-based virus diagnostic testing, creating a need for a timely review. This review first introduces frequent nucleic acid targets for multiplex virus diagnostic tests, then proceeds to a comprehensive and up-to-date overview of multiplex assays that incorporate various detection reactions and readout modalities. The performances, advantages, and disadvantages of these assays are discussed, followed by highlights of platforms that are amenable for point-of-care use. Finally, this review points out the remaining technical challenges and shares perspectives on future research and development. By examining the state of the art and synthesizing existing development in multiplex nucleic acid diagnostic tests, this review can provide a useful resource for facilitating future research and ultimately combating COVID-19.

## 1. Introduction

Since the COVID-19 pandemic began in December 2019, the world has seen over 631 million confirmed cases of infection and nearly 6.6 million deaths [1]. In response, the society has galvanized into actions to combat COVID-19. For example, vaccination against the causative virus, SARS-CoV-2, was miraculously developed in one year after the onset of the pandemic, and since then, ~12.8 billion doses of vaccines have been administered worldwide [1,2]. However, as many regions of the world still suffer from low vaccination rates—coupled with uneven public safety policies and travel restrictions across the globe—several highly transmissible SARS-CoV-2 variants have emerged and caused multiple waves during the pandemic, each time straining the healthcare system [1,2,3,4]. There is also the risk of co-circulation and even co-infection with other respiratory viruses [5]. Thus, nearly 3 years into the pandemic, this global threat has persisted and evolved. 

Throughout the pandemic, the need for rapid, accurate, accessible, and cost-effective diagnostic testing of SARS-CoV-2 based on its viral RNA has been in the spotlight. As the threat from SARS-CoV-2 evolves, the need for diagnostic technologies evolves accordingly, where the detection of SARS-CoV-2 mutations and variants alongside other respiratory viruses has become paramount. To this end, sequencing has been indispensable for tracking genetic mutations in SARS-CoV-2 and other respiratory viruses. The cost, complexity, and lag time of sequencing, however, render it ineffective as a timely diagnostic testing option [6,7]. On the other hand, single-plex diagnostic testing assays that already detect SARS-CoV-2 viral RNA can be expanded to detect multiple targets. In fact, many existing SARS-CoV-2 diagnostic testing assays either detect multiple fragments from one gene or multiple genes to reduce false negative rates due to RNA degradation or false positive rates due to amplification errors [8,9,10,11,12]. Many researchers have also developed multiplexed virus diagnostic testing assays that can detect SARS-CoV-2 variants and other respiratory viruses [13,14,15,16,17]. Some researchers have further developed multiplexed virus diagnostic testing platforms that are amenable for point-of-care (POC) use [18,19,20]. These represent significant advances since the onset of the pandemic, and further advances can be anticipated. 

**Figure 1:**
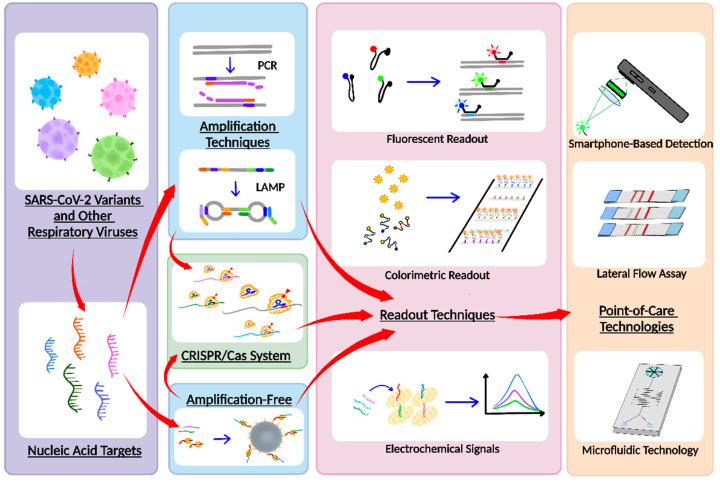
Overall schematic of multiplex nucleic acid diagnostic tests for combating SARS-CoV-2 variants and other respiratory viruses.

We recognize that, in addition to research, a comprehensive review that examines the state of the art and synthesizes existing development of multiplex nucleic-acid-based virus diagnostic tests can be beneficial for guiding future research, propelling further advances, and combating COVID-19. Although there are numerous reviews on various biosensing techniques and technologies for detecting SARS-CoV-2 [21,22,23,24,25], there is only one recent review on multiplex biosensing for SARS-CoV-2 mutation detection [26], and there has yet to be a review focusing on multiplex nucleic-acid-based viral diagnostic tests that also target SARS-CoV-2 variants and other respiratory viruses. In our review, we first briefly introduce the frequent targets for multiplexed virus diagnostic tests. We then provide an up-to-date overview of multiplex virus diagnostic testing assays that incorporate various reaction techniques and detection modalities (Figure 1) while commenting on their performances, advantages, and disadvantages. We also highlight multiplexed virus diagnostic tests that have been implemented within platforms that are amenable for POC use. Finally, we offer perspectives on future research. In doing so, through this review, we hope to facilitate further advances in multiplexed virus diagnostic tests for combating the persistent and evolving threat of COVID-19.

## 2. Definition and Targets for Multiplex Detection

We begin our review by pointing out that we use a liberal definition of multiplex detection to ensure broad coverage of existing diagnostic tests. By traditional definition, a multiplex assay is performed within a single reaction tube (or well) and can simultaneously detect multiple targets that may all be present in a single sample. However, to ensure the breadth of our review, we define “multiplex” as any assay that detects more than one nucleic acid target. Based on this definition, we also include assays that incorporate multiple parallel independent reactions, each typically only detecting one target. These independent reactions can be performed within a single device that houses multiple reaction wells, which can essentially function as a multiplex test.

In this review, targets refer to viral RNA, including SARS-CoV-2 genes and genetic mutations, which lead to SARS-CoV-2 variants, as well as genes of other respiratory viruses. SARS-CoV-2 is an enveloped virus that consists of a lipid membrane and is developed and synthesized by host-cell machinery [3,4]. Four key genes encode key functional proteins [5] and have been commonly targeted: the N gene that encodes the nucleocapsid (N) protein [27], the M gene that encodes the matrix (M) protein [28], the E gene that encodes the envelope (E) protein, and the S gene that encodes the spike (S) glycoprotein [29]. In addition, RNA-dependent RNA polymerase (RdRp) gene and open reading frames (ORFs) regions of the SARS-CoV-2 genome, which enable replication of N, M, E, and S proteins, can also serve as targets for SARS-CoV-2 detection. Among these targets, the N, M, and E genes are more stable throughout the evolution of SARS-CoV-2, while S, RdRp, and ORFs are more liable to undergo a mutation [30]. For example, RdRp is prone to errors due to a lack of a proofreading mechanism, causing 10^−4^ to 10^−6^ mutations per base pair [31]. Such genetic mutations, in combination with homologous recombination, make the viral diversity of SARS-CoV-2 immense and give rise to SARS-CoV-2 variants. As of late October 2022, the World Health Organization has tracked numerous variants, currently designating *Omicron* as a variant of concern (VOC) and monitoring its various subvariants, while listing *Alpha*, *Beta*, *Gamma*, *Delta* as previously circulating VOCs, and several other variants, such as *Epsilon* and *Iota*, as previously circulating variants of interest (VOIs). Genetic mutations in SARS-CoV-2 variants can potentially cause false negatives, further driving the need for multiplex testing. For example, as the S gene harbors frequent mutations, single-plex assays that target the S gene must be regularly validated to avoid false negative results [30]. Finally, similar symptoms are shared between SARS-CoV-2 and other respiratory viral pathogens, such as non-SARS-CoV-2 coronavirus, influenza virus, adenovirus, rhinovirus/enterovirus, and parainfluenza virus. In response, the United States Center for Diseases Control and Prevention and the Food and Drug Administration have updated the testing guidelines to co-test for SARS-CoV-2 alongside Influenza A/B. As of the writing of this review, however, simultaneous testing of SARS-CoV-2 with other respiratory viruses currently remains infrequent, typically limited to Influenza A/B and respiratory syncytial virus (RSV) [32,33,34,35]. 

## 3. Multiplex Viral RNA Detection and Amplification Methods

To date, researchers have reported a variety of multiplex virus diagnostic testing assays that can distinguish wild-type (WT) SARS-CoV-2 genes from VOCs, in addition to co-detection methods with other respiratory viruses. Among these viral RNA detection reactions, polymerase chain reaction (PCR) remains the most common, but isothermal reactions, particularly loop-mediated isothermal amplification (LAMP) and recombinase polymerase amplification (RPA), have gained significant traction since the onset of the COVID-19 pandemic. Moreover, clustered regularly interspaced short palindromic repeats (CRISPR)-based reactions are rapidly emerging as well among other unique assays. In this section, we briefly introduce the various assays that utilize these reactions.

### 3.1. PCR

**Figure 2:**
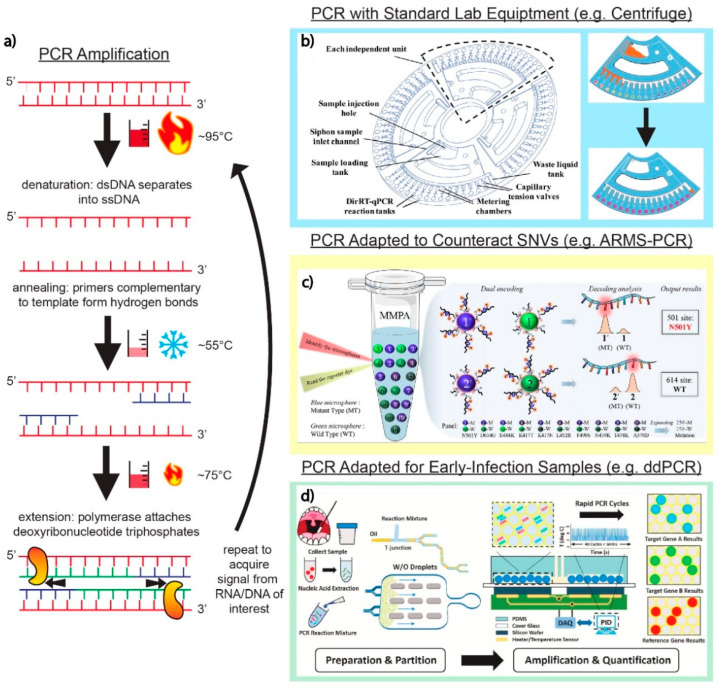
PCR techniques and applications. (**a**) Basic schematic of the core PCR amplification steps. The dsDNA containing the targets of interest are denatured into two separate ssDNA strands. Hybridization of primers complementary to target sequences of interest signals DNA polymerase to amplify ssDNA targets through thermocycling. (**b**) Adaptation of a standard laboratory centrifuge into a fully customizable disc that allows for PCR into 64 independent direct RT-qPCR across four independent loading units. Adapted with permission from Ref. [42]. Copyright 2020, Royal Society of Chemistry. (**c**) The utilization of fluorescent microspheres (MPA enhanced ARMS-PCR platform) for the amplification of fluorophores on primers targeting genes of interest in the detection of SARS-CoV-2. Adapted with permission from Ref. [48]. Copyright 2022, Elsevier. (**d**) Decreasing the signal-to-noise ratio of genomic target to background interference using the ddPCR platform. As shown in this platform, a series of microfluidic channels create individual droplets, which can then be separated into individual bioreactor units on a silicon chip. Adapted with permission from Ref. [60]. Copyright 2021, Elsevier.

Reverse transcription-polymerase chain reaction (RT-PCR) is the gold standard for SARS-CoV-2 detection due to its reliability, high sensitivity, and specific results. PCR, as outlined in Figure 2a, involves the denaturing of double-stranded DNA (dsDNA) into single-stranded DNA (ssDNA). From there, primers anneal to the nucleic acid sequences of interest, and DNA polymerase synthesizes a new nucleotide string. Repeated thermal cycles amplify targets over time. The current benchtop PCR instruments (e.g., cobas^®^ 6800 System (Roche Diagnostics, Basel, Switzerland)) utilize a variety of SARS-CoV-2 master mixes (e.g., Takara One Step PrimeScript III RT-qPCR kit (Takara Bio, Kusatsu, Japan)). A particularly notable PCR-based test is the BioFire^®^ Respiratory Panel 2.1. This commercial test can detect SARS-CoV-2 and an additional 21 viral and bacterial pathogens and has been clinically evaluated [36,37]. The critique of PCR-based methods predominantly centers around the need for bulky and delicate thermal cyclers that require professional calibration and maintenance. Issues such as these have inspired new technological developments in SARS-CoV-2 multiplex assay development.

#### 3.1.1. Multiplex PCR Using Modified Benchtop Equipment

Traditionally, multiplex benchtop PCR assays have been designed to observe specific targets in WT SARS-CoV-2 composition or VOCs and VOIs closely related to WT targets for transmission data in an infected population. For detecting the WT strain in early 2020, multiplexing key regions, such as N1/N2, E, RdRp, and S, among others, was popular [38]. Since the emergence of VOCs and VOIs, ORF1a and S genes were targeted for their detection [39,40,41]. Other researchers have developed PCR-based assays to facilitate the detection of SARS-CoV-2 in conjunction with influenza A/B [13,14,15,16,17], RSV, human metapneumovirus, adenovirus, human rhinovirus, and parainfluenza viruses [13].

Unconventional and creative ways of performing the steps of the PCR process, particularly thermocycling and mixing, have emerged in several works reviewed here with widely available, low-cost equipment. A direct reverse-transcription quantitative PCR (dirRT-qPCR) assay developed by Minghui Ji et al. (Figure 2b) utilizes an engineered centrifuge, built with an optical detection unit and a thermocycler, rotating a disc with four loading chambers for one-step clinical sample loading. As the centrifugal force overtakes the sample, it is pulled down to compartments along the edge of the disc for a total of 64 different RNA samples that could theoretically be tested. The injection-to-answer platform is around three times faster than a benchtop RT-PCR, with no less than 99% agreement in detecting SARS-CoV-2 and Influenza A or B RNA against a RT-PCR control [42]. 

In many laboratory settings, water baths are commonplace; inspired by them, Chen et al. developed a rapid water bath PCR, lateral flow assay (LFA) platform. An automated mechanical set-up shuttled the reaction tubes between water baths utilizing a dual-stepper motor system programed to periodically expose the reaction tubes to varying degrees of warmth between the baths (set at 98 °C and 53 °C). This technology can enable 40 cycles of amplification in 30 min, faster than conventional RT-PCR. The low-cost set-up can sustain higher throughput by utilizing the entire volume of the bath [43]. In conventional RT-PCR, the thermal cycler ramping rates may result in multiple days between sample collection and viral detection, prolonging test results for patients.

#### 3.1.2. Single Nucleotide Variant Distinguishing Multiplex PCR

SARS-CoV-2 VOIs and VOCs contain multiple single nucleotide variants (SNVs) that aid the virus in enhanced transmission and immune system evasion. Patient samples may produce false negatives due to one SNV when analyzed with single-plex, RT-PCR techniques. This was speculated early during the SARS-CoV-2 pandemic with an SNV in the N gene [44,45] and persists with ultramodern VOCs, such as the *Delta* variant [46]. Amplification refractory mutation system (ARMS) PCR technology is a powerful method to discriminate point mutations. ARMS-PCR has been validated as an advantageous way to detect variants and mutations during the pandemic [47,48]. Using a tetra-primer ARMS-PCR assay, Wang et al. were able to separate a WT strain of SARS-CoV-2 from two separate SNV versions of the virus. When allele-specific primers are perfectly complementary, PCR products are synthesized and amplified. A biotin or digoxin label was attached on either mutant or wild-type PCR products in their assay, and their customized LFA platform was able to detect if the patient had a positive result, and more specifically, what type of result they had (WT or one of two SNVs), using quantum dot nanobeads covered in streptavidin or digoxin antibody. This technology can be read with a simple UV light and can yield results in less than 2 h with their USD 150 two-channel fluorescence readout module [47]. 

A team led by Zhong et al. developed a microsphere-phase amplification (MPA) enhanced ARMS-PCR platform, using primers attached to microspheres to locate and complement single-nucleotide SARS-CoV-2 variants, limiting mutual interference between different target primers. This technology is highlighted in Figure 2c. Uniquely, the fluorescence of the reporting probes and the microspheres on which amplification occurs produces a unique acquisition signal for each target. For high-multiplex reactions, this effectively diminishes fluorescent channel availability constraints. Up to 500 different targets can be theoretically distinguished, despite the group only analyzing 10 VOIs. The use of several primers across multiple variants increases the probability of non-specific amplification, decreasing specificity and sensitivity. Multiplex MPA overcomes the risk of false positives frequently and consistently observed in ARMS-PCR techniques [48]. An article published by Wang et al. also describes SNV detection using *Pyrococcus furiosus* Argonaute (PfAgo) in a nucleic acid detection method platform. When PfAgo, a guide DNA strand, and a molecular beacon are mixed, the amplified RNA strands from RT-PCR interact with PfAgo, cleaving the target DNA to generate a guide DNA strand. This action allows a secondary cleavage to occur and generates a fluorescent signal by removing the quencher from the fluorophore on the beacon. In theory, only point mutations that match the input guide DNA on the PfAgo complex will amplify a signal, initiating a cascade of cleavages for downstream signal acquisition [49]. 

#### 3.1.3. Early Detection Multiplex PCR

Early detection using PCR for VOCs is challenging; the key biomarkers are not fully developed during the early onset of SARS-CoV-2 infection, circulating in concentrations typically undetectable during acute infection. This information can be captured utilizing digital PCR (dPCR), a system dedicated to randomizing molecule distribution into multiple partitions for absolute quantification, without previous calibration [50]. Each partition acts as a microreactor for PCR amplification before detection.

Wastewater samples are a unique method of monitoring infection levels on a community scale instead of the traditional individual-to-individual diagnosis. New PCR methods are required to analyze wastewater samples, as trace signal is lost in the complex sample matrix for conventional RT-PCR to analyze and is often never found due to amplification inhibitors [51,52]. The dPCR amplification mechanism has enabled the use of wastewater for SARS-CoV-2 detection, giving near real-time population data from several countries spanning the globe [53,54,55]. Because urine and feces contain fragments of the SARS-CoV-2 genome, this allows for high spatial and temporal resolutions of VOCs spreading through a community-level population by way of one, individual sample. Eliminating hundreds to thousands of collected samples limits costs and economizes testing kits. One such technology is demonstrated by Boogaerts et al., using dPCR to detect ultra-low concentrations (0.3 to 2.9 RNA copies/μL) of four VOC targets from wastewater samples. The dPCR process can withstand heavy metals or organic matter that could limit amplification efficiency in conventional RT-PCR. Over 26,000 partitions in the technology used by the team can maximize discrepancies in mutated and WT SARS-CoV-2 RNA while avoiding larger volumes that emit intense background noise from inorganic and organic matter. Although the complex matrix of wastewater was mitigated through centrifugation, filtration, and dPCR segmentation, the primer sets for detection of N501Y mutations, for instance, were found to be potentially less sensitive and could reveal false negatives [56]. 

Droplet digital PCR (ddPCR) eliminates the complexity of PCR by incorporating a droplet generator that keeps uniformity in sample distribution, maintaining droplet size on the nL to pL scale. This improves the signal-to-noise ratio, allowing this technique to be rather valuable for the early detection of SARS-CoV-2 from biological samples. A quadruplex ddPCR targeting system developed by Nyaruaba et al. demonstrated the detection of SARS-CoV-2 complementary DNA (cDNA) at ultra-low concentrations, detecting all cDNA samples between one and three copies/reaction among the cDNA tested [57]. Other works have demonstrated that by utilizing droplet-generating PCR systems (e.g., QX200 ddPCR System (Bio-Rad, Hercules, CA, USA)), a triplex can usually be achieved [58], with others demonstrating a multiplex of six [59]. The droplet digital, rapid PCR method by Yin et al. (Figure 2d) separates unique primers into individual “bioreactor” droplets with specially designed heater arrays that lead to rapid amplification on a microfluidic platform. The group demonstrated a 2 min qualitative readout time at a sensitivity of five copies/reaction for the N, ORF1ab, and RNase P genes of SARS-CoV-2 [60]. Rapid PCR thermocycling using the ddPCR method in this application dramatically improves the test-to-result times needed for patients not to expose themselves to others, with the sensitivity to observe early stage infectious RNA material.

### 3.2. LAMP

Reverse transcriptase loop-mediated isothermal amplification (RT-LAMP) assays are an isothermal alternative to conventional RT-PCR, eliminating the need for time-consuming thermal cycling while simplifying the assay design requirements. LAMP assays use four to six primers and a strand invading polymerase (*Bst*), annealing and separating the target DNA. The newly formed sequence forms self-hybridizing loops on either end, creating a dumbbell structure from which amplification occurs during 65 °C incubation.

With simple equipment requirement and accuracy comparable to PCR, LAMP assays using commercial detection kits for SARS-CoV-2, typically with a colorimetric readout in a microcentrifuge tube have rapidly become popular. WarmStart SARS-CoV-2 Rapid Colorimetric LAMP Assay Kit (New England Biolabs, Inc., Ipswich, MA, USA) incorporates RNA purification of either nasal swabs or saliva into the kit’s workflow, targeting both the N and E genomic regions, with a color change from pink to yellow if amplification occurs. SARS-CoV-2 Colorimetric ReadiLAMP™Kit (ThermoFisher Scientific, Waltham, MA, USA) incorporates saliva or a nasopharyngeal sample directly as assay input, using viral lysis to allow amplification without RNA extraction. 

In the midst of the enthusiasm for this isothermal NAAT, challenges in multiplex LAMP detection persist due to the substantial number of primers required for a single reaction. Multiple primers can unintentionally interact and experience non-specific binding and dimer interactions that may impact assay sensitivity and lead to false positives. Additionally, single-point mutations during amplification and incorporating a sample collected at the point of care without prior RNA isolation are further challenges faced in multiplex LAMP assays [61].

#### 3.2.1. Multiplex LAMP Using Modified Benchtop Equipment

To date, researchers have reported several benchtop multiplex LAMP assays targeting multiple SARS-CoV-2 genes. For example, Jang et al. designed three sets of LAMP primers that could be combined together for a triplex assay for detecting the RdRp, E, and N genes [62]. In an effort to improve the reliability and robustness of detection, Juscamayta-López et al. investigated dimer interaction between primer sets to optimize three sets of LAMP primers for a triplex RT-LAMP reaction that detects the RdRp, M, and ORF1ab genes with as few as 100 copies/µL, using pre-processed RNA samples [63]. The attention to detail in primer sequence design can be applied to any number of readout modalities, including fluorescent or electrochemical. Both assays are successful in optimizing the amplification time and have high certainty, but they require RNA isolation to be performed within a lab utilizing specialized and expensive reagents.

Despite the best design efforts, multiplex primer sets can still experience non-specific binding. To find a positive signal among the background noise of amplification byproducts, oligonucleotide strand exchange was utilized by Bhadra et al. as a reporting technique with high sensitivity. Fluorescent reporters, coupled with opposing quencher moieties for multiplex detection, bound to highly specific “toehold” regions with lower mutation rates within the amplified target sequence, subsequently displacing the quencher molecule. This technique can be enabled to detect nucleic acid sequences in RNA with as few as 3 copies/µL from a pre-processed saliva sample. This high sensitivity offsets the impact of dimer interactions and lower amplification rate, but it also has lower specificity than a standard PCR test [64]. 

#### 3.2.2. RNA Extraction-Free Multiplex LAMP 

Dong et al. developed an RNA extraction-free RT-LAMP, starting with a nasal swab sample suspended in RNase-free water and incubated for 10 min at 95 °C to inactivate, followed by direct RT-LAMP detection. Highly specific, real-time detection was accomplished using a high-fidelity DNA polymerase mediated (HFman) probe. HFman probes have fluorophore and quencher moieties, as well as a complementary sequence to the dumbbell structure created using “loop” primers during the initial amplification stage of the LAMP assay. During the exponential amplification phase, the HFman probe is cleaved by a high-fidelity DNA polymerase, releasing the quenching moiety and providing a real-time fluorescence readout. Multiple detector fluorophores can be used simultaneously; this work distinguished the ORF and E genes for both wild-type and *Alpha*, *Delta*, *Gamma*, and *Omicron* VOCs, with 100% specificity [65]. 

In another advance toward high-certainty POCT, Nguyen et al. adapted the RT-LAMP assay to a 3D-printed microchip with preloaded LAMP solution, freeze-dried enzymes (*Bst* DNA polymerase and reverse transcriptase), and primers, which can be loaded directly with 2 µL of nasopharyngeal swab sample. The microchip is inserted into a “portable genetic analyzer”, a purpose-built, battery-powered diagnostics tool combining a smartphone-based controller, heater, and servo motor for vibration-assisted cell lysis and enzyme mixing. Using a direct lysis buffer during an initial 10 min inactivation period at 95 °C, thereby avoiding RNA purification, this POCT device provides a fluorescent readout time within 75 min, targeting the As1e, N, and E genes within the SARS-CoV-2 conserved regions of the genome. This fluorescent readout can be detected by a smartphone, and the results can be stored via a cloud-based app, making the portable genetic analyzer an entirely self-contained LAMP kit [66]. 

### 3.3. RPA

One of the most widely utilized assays in developing POCTs for SARS-CoV-2 is reverse transcriptase recombinase polymerase amplification (RPA), another isothermal (37–42 °C) amplification technique. During RPA, recombinase proteins form complexes with a target primer and the double-stranded DNA target. At this point, the double strand is separated into single strands, stabilized by single-strand binding proteins, for amplification. Compared to conventional benchtop assays, RPA does not require an expensive thermocycler or bulky dedicated equipment, as demonstrated with conventional PCR assays [67]. RPA can be coupled with different detection techniques, including CRISPR-based technology with fluorescence or colorimetric readouts. Some of these technologies will be discussed in the CRISPR-based assays section.

Because RPA is an isothermal amplification technique, it does not require a denaturation step to separate double-stranded DNA and only needs two primers, while LAMP requires more primers and a higher operating temperature [11]. With this in mind, RPA has many opportunities to be developed and applied to POC applications. Using easily available benchtop equipment, Cherkaoui et al. performed a clinical validation of a one-pot multi-gene RT-RPA assay targeting the E and RdRp genes and reported multiplex detection of 11 SARS-CoV-2 lineages (e.g., *Alpha*, *Beta*, *Delta*, and *Omicron*) with a sensitivity of 96% and specificity of 97%. They additionally explored a method to translate the time to threshold from RT-RPA results to viral load equivalents, allowing a semi-quantitative detection method [68]. 

Without the need for denaturation, RPA can be carried out directly in complex matrices, such as urine and stool samples. By contrast, the traditional PCR assays perform poorly due to the presence of inhibitors, such as hemoglobin and heparin. High genomic DNA concentrations were reported to affect both the PCR and RPA assays. Further RPA limitations include a paucity of available RPA kit manufacturers—a situation similarly faced by LAMP—or purpose-built software for designing RPA primers. Other than reagent shortage issues, which are common among NAAT assays, custom-built toolkits also lead to a higher cost per RPA reaction [11,68]. 

To avoid more complexity and the increase in reaction time due to the addition of a CRISPR-based assay, Cherkaoui et al. developed a rapid molecular diagnostic technique utilizing one-pot RT-RPA to simultaneously detect two gene targets of SARS-CoV-2. Two alternative readouts were designed, achieving a low LOD of 9.5–130 copies/reaction within 20–30 min. The first readout is a real-time fluorescence analysis using exonuclease cleavage of the fluorescence probes, and the second readout is an end-point dipstick readout using a lateral flow assay with nanoparticle labels. Even though an expensive portable fluorescence reader and a personal computer were still required for the real-time fluorescence readout, the group also explored the use of low-cost hand warmers for providing a required temperature of around 37 °C. Additionally, a smartphone could be utilized for monitoring and interpreting the results, enabling worldwide accessibility in a variety of environments [69].

### 3.4. CRISPR-Based Assays

**Figure 3:**
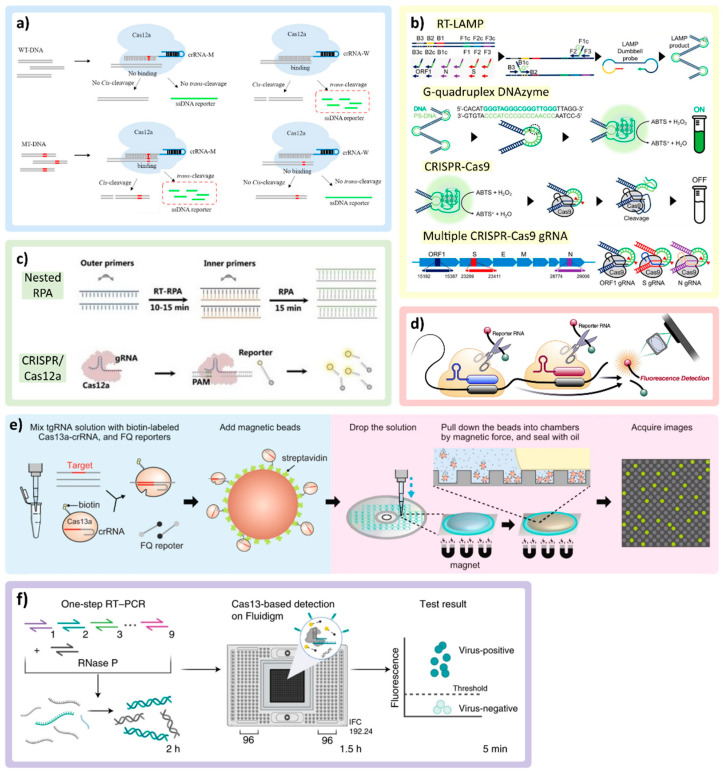
CRISPR/Cas technologies and multiplexing applications. (**a**) Demonstration of cis-cleavage and trans-cleavage of CRISPR/Cas mechanism, as crRNA-M recognizes the mutant DNA (MT-DNA). Adapted with permission from Ref. [70]. Copyright 2022, Elsevier. (**b**) Multiplex colorimetric assay triggered by LAMP (ON signal) and eliminating false positives by using a CRISPR/Cas9 system (OFF signal) to detect VOCs. Adapted with permission from Ref. [77]. Copyright 2022, American Chemical Society. (**c**) The combination of nested RPA and CRISPR/Cas12a in the same reaction pot. Adapted with permission from Ref. [75]. Copyright 2022, Wiley-VCH. (**d**) Multiple crRNAs targeting different regions of SARS-CoV-2 RNA with single reporter RNA type to improve the sensitivity of the assay. Adapted with permission from Ref. [12]. Copyright 2021, Elsevier. (**e**) The magnetic beads conjugated with CRISPR/Cas complex triggered by target RNA and pulled down by the magnetic force into the microchamber, concentrating the reactions. Adapted with permission from Ref. [74]. Copyright 2022, Springer Nature. (**f**) RT-PCR CRISPR-based diagnostic and microfluidic panel, mCARMEN, for parallelizing nucleic acid detection, utilizing a commercial chip Fluidigm, which can identify up to 21 viruses. Adapted with permission from Ref. [71]. Copyright 2022, Springer Nature.

Clustered regularly interspaced short palindromic repeat (CRISPR)/ CRISPR-associated (Cas) systems are utilized in many multiplex detection techniques in both amplified and unamplified nucleic acid detection. CRISPR-based systems have a specific recognition mechanism enabled by the guide RNA (gRNA or crRNA) forming a complex with the Cas enzyme. As shown in Figure 3a, the binding between crRNA and the target-activated CRISPR effector protein (Cas), at room temperature, enables cleavage activity on the target (*cis*-cleavage) and subsequent non-specific activity on non-target (*trans*-cleavage) [70]. For example, upon binding to ssDNA or ssRNA, the *trans*-cleavage mechanism can release a fluorescence reporter from the quenched fluorescence probe [9,12,20,70,71,72,73,74,75,76]. Different CRISPR-Cas systems have been utilized for multiplex SARS-CoV-2 detection, including the Cas9 system (recognizes dsDNA) [77,78], Cas12 (recognizes both ssDNA and dsDNA) [9,71,72,75,76,79], and Cas13 (recognizes ssRNA) [12,20,71,74,80,81]. Additionally, amplification techniques, such as RT-PCR [9,71,72,76], RT-LAMP [73,77,79], and RT-RPA [9,20,75,78], are often combined with CRISPR-based detection to enhance the limit of detection (LOD) in viral detection assays. 

#### 3.4.1. Multiplex CRISPR/Cas Assays with Nucleic Acid Amplification 

Benchtop assays can be considered as critical initial steps for designing and optimizing the CRISPR-Cas system (e.g., designing crRNA and selecting the Cas protein). One challenge is the requirement for the protospacer adjacent motif (PAM) present on the target strand, which typically serves as the binding signal for the Cas protein. The possible mismatch of PAM on the target sequence can affect the cleavage rate; this is especially relevant for multiplex detection. Liang et al. addressed this issue by developing a universal system that introduces PAM near the target mutation sites through PCR primer design, allowing mutations detection even if the target sequence did not originally contain the PAM adjacent to the mutation sites. By coupling RT-PCR with the CRISPR-Cas12a genotyping assay, SARS-CoV-2 variants (*Alpha*, *Beta*, *Gamma*, *Delta*, *Kappa*, *Lambda*, and *Epsilon*) could be detected with high sensitivity, targeting signature mutations [76]. 

Utilizing primer-enabled detection, similarly, Song et al. developed a multiplex colorimetric assay triggered by LAMP amplification with false positives eliminated by using a CRISPR/Cas9 system to detect specific VOCs (*Delta* and *Omicron*). The primer sets were designed around the target genes for both WTs and mutations (MT) with the addition of G-quadruplex complementary sequence with phosphorothioate modification to lower the melting temperature. As represented in Figure 3b, as the LAMP proceeds in the presence of SARS-CoV-2 RNA, more G-quadruplex DNA/PS-DNA duplexes are produced within the amplicon products, which will interact with hemin, turning on colorimetric signal in the presence of H_2_O_2_ through peroxidase activity. To double check specific mutation genes, CRISPR/Cas9 with crRNA specifically designed to recognize the target genes (WT or MT) will cleave the G-quadruplex DNAzyme, which turns off the colorimetric signal. For multiplexing, the on/off colorimetric signals of multiple genes tested in parallel were derived to determine the VOCs [77]. 

Another popular amplification technique used with the CRISPR/Cas system is RT-RPA [9,20,75,78]. An assay developed by Xiong et al. uses modified RPA primers to produce biotin-E gene and digoxin-ORF1ab gene amplicons from SARS-CoV-2 RNA in a single assay. Two test lines on the same paper strip are localized either with streptavidin (which binds to biotin) or anti-digoxin antibody (which binds to digoxin). To enable colorimetric detection, the crRNA designed for each target is modified by adding the constant scaffold region, which contains a binding site for recruiting gold nanoparticle (AuNP)-DNA probes. If the target is present, the AuNPs bind to crRNA of the CRISPR/Cas9 complex and aggregate on the paper strip along the tested lines, becoming visible to allow colorimetric detection [78]. The combination of nested RPA (RT-RPA for the general target and RPA for specific genes) and the CRISPR/Cas system to detect up to eight viruses is also demonstrated in Figure 3c reported by Lui et al. [75]. 

#### 3.4.2. Amplification-Free Multiplex CRISPR/Cas Assays

To reduce the assay time, steps, complexity, and possible contamination, some groups directly utilized the CRISPR/Cas systems without nucleic acid amplification [12,74,76,80]. Commonly, the nucleic acid amplification step allows assays to achieve high sensitivity and specificity. As the CRISPR technology can provide specificity through crRNA, the challenge faced by amplification-free CRISPR assays is sensitivity [82], which has been tackled by using alternative sensitivity enhancement techniques by several research groups. Fozouni et al. combined multiple crRNAs targeting different regions of SARS-CoV-2 RNA with Cas13a. The same reporter RNA is cleaved upon activation to improve the sensitivity of the assay, as shown in Figure 3d. It also allows better accessibility, as the complexity of the device and assay can be reduced due to CRISPR/Cas reactions enabling operation at room temperature [12]. 

Different molecular manipulation techniques (e.g., magnetic fields, electric fields) may be employed to improve assay sensitivity and/or enable automated assays. For example, Shinoda et al. used streptavidin-coated magnetic beads to capture biotin-labeled LtrCas13a (Cas13a from *Leptotrichia trevisanii*)-crRNA complexes that form hybrids with pre-incubated -target RNA as means to improving assay sensitivity (Figure 3e). Moreover, multiple crRNAs are designed to target mutations in the SARS-CoV-2 S gene. The mixed reagents are added on the custom digital chip, and the neodymium magnet is placed underneath to generate the magnetic force, pulling the magnetic beads down into a microchamber, concentrating the reactions. This platform allows a ~50-fold increase in sensitivity and can differentiate VOCs, including *Alpha*, *Delta*, and *Omicron*, within 9 min [74]. Despite the rapid assay time, the custom-built CD-based microchamber device and dispensing robot-based instrumentation employed in this platform could pose challenges in terms of cost and accessibility. Magnetic beads can also be used to extract the RNA target from the complex matrix in both amplification-free and pre-amplification formats. Lui et al. used two types of coated magnetic beads for targeting non-specific RNA of general viruses (silicone-coated) and targeting the poly-A chain on the SARS-CoV-2 RNA (poly-T-oligonucleotide-coated) combined with multiplex nested RPA [75]. 

Another technique used to extract RNA and manipulate the reaction was demonstrated by Ramachandran et al. using an electric field gradient. Isotachophoresis, an electrokinetic microfluidic technique, was utilized by capturing the target ions between two buffers (high-mobility leading buffer and low-mobility trailing buffer). This process allows for RNA extraction and co-focusing Cas12-crRNA, reporters, and targets to accelerate the reaction under the electric field. This technique can achieve the detection of SARS-CoV-2 N, E, and human RNase P genes within ~30 min [73]. Finally, another research team led by Li et al. utilized an electric field CRISPR/Cas system to regulate electrochemical reactions combined with Cas13a [80]. 

#### 3.4.3. Modified Platforms for Multiplex CRISPR/Cas-Based Assays

CRISPR-based multiplexing assays may be conducted in a one-pot reaction targeting ~2–8 target genes at once [75,78]. However, to improve multiplexing capabilities, many groups utilized CRISPR/Cas reactions running separately in parallel with crRNAs specifically designed to target mutation genes using different platform designs [9,70,71,77]. For example, Welch et al. developed a combined RT-PCR CRISPP-based diagnostic and microfluidic panel, mCARMEN. This technology parallelizes nucleic acid detection, utilizing the commercial Fluidigm microfluidic cartridge and the commercial Fluidigm Biomark instrument to identify up to 21 viruses, including 6 variants of SARS-CoV-2 (Figure 3f). The Fluidigm microfluidic cartridge houses spatially separated samples and reagent wells and a reaction chamber chip with thousands of reaction chambers, which are connected via individual units of integrated fluidic circuits. Upon manual loading of the samples and fluorescence-based assay reagents into designated wells, the Fluidigm instrument, which incorporates fluidic control, thermal control, and optics, moves the samples and assay reagents through individual integrated fluidic circuits to the reaction chamber chip and performs mixing, reaction incubation, and fluorescence signal acquisition. The quantitative measurement is achieved by utilizing different reaction kinetics and enzymatic activities of multiple CRISPR/Cas proteins and three fluorescence channels. Nevertheless, the manual sample and reagent loading to the Fluidigm was still required, which contributed to the up to 5 h assay time [71]. Other platforms and techniques that are utilized to facilitate the assays with CRISPR/Cas systems include lyophilization of CRISPR/Cas reagents to simplify the assay for the end user [20,72], microfluidic chips [12,72,73,75], lateral flow assay [20,70,78], and smartphone-based detection [9,12,77]. 

### 3.5. Other RNA Detection Methods

In addition to conventional nucleic acid amplification reactions that we described previously, alternative multiplex virus diagnostic testing assays have also been developed to directly identify the viral targets present to reduce the complexity, cost, and possible contamination [12,83,84,85,86,87]. For example, a magnetofluorescent bio-platform for direct detection of SARS-CoV-2 is demonstrated by Zayani et al. Using a nasopharyngeal swab sample directly, the ORF1a and S gene probes are tethered to magnetic beads with a biotin/streptavidin linkage to extract the RNA target. The RNA is separated and then hybridized with horseradish peroxidase (HRP)-conjugated reporter probes. An HRP-catalyzed fluorescence readout is generated in less than 5 min by adding hydrogen peroxide and o-phenylenediamine. This technique showed acceptable sensitivity and high selectivity with a LOD of 1000 copies/μL and was able to distinguish SARS-CoV-2 RNA from related viruses, such as Hepatitis C, West Nile, and measles [87]. This reaction is rapid and simple, as it obviates amplification and all associated sample preparation steps.

## 4. Readout Modalities 

### 4.1. Fluorescence

**Figure 4:**
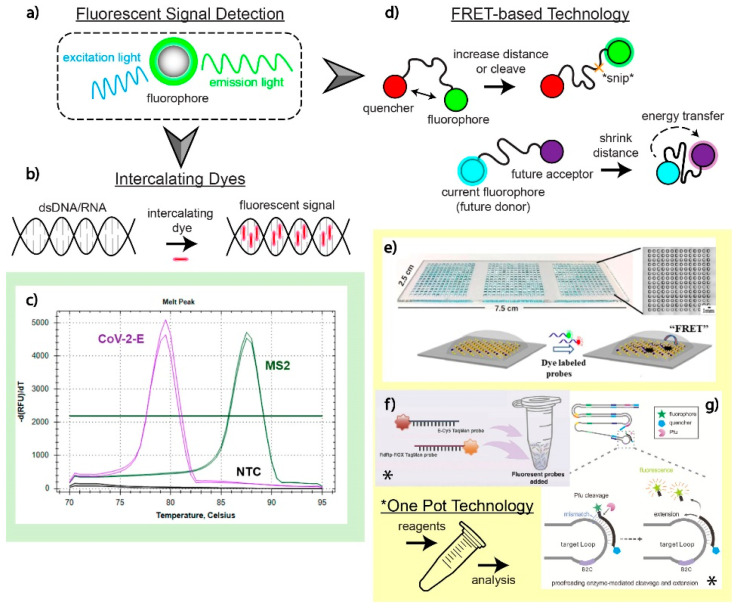
Fluorescence readout and applications. (**a**) Illustration of how fluorescent signals are generated to detect SARS-CoV-2 nucleic acids of interest. Fluorescent optical detection involves a fluorophore emitting a signal at a particular wavelength from an initiating, excitation wavelength. (**b**) Intercalation of dyes sandwiched between the hydrogen bonds of complementary nucleic acid sequences. (**c**) Analysis of melting curve profiles to detect and distinguish two targets in a multiplex assay utilizing intercalating dyes as a fluorescent signal. Adapted with permission from Ref. [89]. Copyright 2021, the Authors. (**d**) FRET-based fluorescent detection has two distinct mechanisms: increasing the distance between a fluorophore and the quencher or decreasing the distance between two fluorophores to allow for energy transfer from one fluorophore to another. (**e**) A MoS_2_ nanosheet-modified DMA platform designed to detect SARS-CoV-2 genomic material on the nM scale using a FRET-based optical detection method. This multiplex test enabled the detection of SARS-CoV-2 genetic material alongside HIV. Adapted with permission from Ref. [90]. Copyright 2020, American Chemical Society. (**f**) A one-pot OPIPE assay utilizing pre-coated tubes for the detection of RdRp and E genes. The TaqMan probes used have different fluorophores that enable fluorescent detection after cleavage during the RT-PCR amplification process. Adapted with permission from Ref. [102]. Copyright 2021, Elsevier. (**g**) Utilization of the Proofman enzyme, a protein that recognizes SNVs, which can cleave fluorophores away from restrictive quenchers on a primer specifically targeting mutations. This set-up is performed in a singular pot for multiple targets of interest. Adapted with permission from Ref. [103]. Copyright 2021, Elsevier.

Fluorescent technology as an optical detection method for viral nucleic acid amplification works by exposing fluorescent molecules (fluorophores) to a particular wavelength of light. This causes electrons in the molecule to transition from a ground to an excited state and quickly back to the initial ground state position, releasing photons at a specific wavelength that can be observed and quantified (Figure 4a). Fluorescent tagging for optical detection is reliable, has high specificity at the molecular level, and is extremely sensitive.

#### 4.1.1. Intercalating Dyes

Intercalating dyes are popular for detecting amplicons due to their simplicity. When detecting nucleic acid targets, these dyes are not specific and will intercalate in dsDNA present in the sample. Theoretically, only nucleic acids of interest will be amplified continuously, leaving off-target regions unamplified and less concentrated in the working solution. A signal will be captured during the amplification event because intercalating dyes bind between the complementary base pairs of DNA, as shown in Figure 4b. This process is risky, however, and off-target nucleic acids being amplified will lead to false interpretations of the test. Typically, a melting curve analysis is performed after intercalation occurs to detect the differences in the genomic strands of amplified nucleic acid targets. The nucleic acid composition, length, and other factors lead to a unique melting curve profile. Metal ion indicators are also used in place of intercalating dyes for SARS-CoV-2 detection [88]. 

The work by Oscorbin et al. demonstrates the ability to detect a duplex of bacteriophage MS2 RNA and the E gene of SARS-CoV-2 using intercalating dye SYTO-82. After LAMP amplification of these targets of interest and the addition of SYTO-82, a melting curve analysis was performed and demonstrated an exceptionally low LOD for the E gene (20 copies/reaction) in the duplex detection with MS2 [89]. The melting curve from this study is shown in Figure 4c and demonstrates that multiplex detection can be achieved without any specificity of the dye. The overlapping of melting peaks is particularly challenging for proper analysis post-amplification. This poses a great difficulty to multiplex several different SARS-CoV-2 genes of interest, as the minute changes to the genome do not yield a dramatically different melting curve. This process is not well suited for SNV analysis and distinction. Nonetheless, multiplexing could be achieved in this format to distinguish a SARS-CoV-2-related illness from other potential pathogens, assuming enough differences can distinguish themselves on a melting curve.

#### 4.1.2. FRET-Based Techniques

Fluorescence resonance energy transfer (FRET) via scorpion probes, molecular beacons, or other platforms are unique ways to gather fluorescent signal from different amplification processes (Figure 4d). Nanoscale molecular interactions are amplified by FRET, allowing for higher resolution images with high specificity for targets of interest. To minimize reagent consumption and assay time while maximizing sensitivity and multiplex capacity, Oudeng et al. developed a molybdenum disulfide (MoS_2_) nanosheet-modified dendrimer droplet microarray (DMA), as shown in Figure 4e. The dendrimer-based DMA platform allows for the fixation of multiple nanomaterials on a large surface area substrate to increase biosensing capacity. Nanosheets made of MoS_2_ are adsorbed on dendrimers to house the individualized FRET assays. When the target nucleic acids come in proximity to the sensing probes on the MoS_2_ sheet, these probes will become detached from the surface, subsequently increasing the distance between the fluorophore and the quencher, enabling fluorescent target detection. Although this technology was dedicated to HIV nucleic acid detection, the group also investigated the detection of the ORF1ab and N gene of SARS-CoV-2 on the same platform with nM resolution [90].

TaqMan probes, a common probe in nucleic acid detection, are a simple way to read multiplex signals for multiple targets of interest during PCR amplification [16,91,92]. In a study by Dharavath et al., the group was able to fabricate a one-step, singular-pot RT-PCR assay with a computer analysis tool that eliminated bias in reading raw qRT-PCR data. In their set-up, fluorescent molecules (FAM, HEX signal for N1+N2 genes, control) are attached to primers that are complementary to nucleic acid sequences of interest during the annealing phase of PCR [93]. When the polymerase builds dsRNA during the extension phase, the primer is hydrolyzed to make room for the polymerase to attach new nucleoside triphosphate molecules, releasing the reporter from a quencher to fluoresce. Although the assay does not distinguish N1 from N2 detection, it is possible to amend different probes onto specific primers depending on the fluorescent channels at disposal. The built-in qPCR analyzer tool runs an unbiased algorithm analyzing fluorescent signals from the test, complete with a graphical user interface for simplified control–operator interaction (2 h, USD 3 per test) [93].

Whereas TaqMan probes are more popular in PCR-based amplification, molecular beacons are more popular in LAMP-based SARS-CoV-2 assays. Typically, hairpin-shaped μRNA beacons undergo hybridization to a target RNA sequence, where they subsequently unfold and separate the quencher from the fluorescent dye. In other applications, two beacons (an acceptor and a donor) will recognize a target RNA strand and nucleotide sequence nearby to allow a higher resolution, FRET-based signal. Research by Sherrill-Mix et al. developed LAMP-BEAC, a system using a molecular beacon to detect four different regions of interest along the SARS-CoV-2 genome [19]. The quadruplex design has the adaptability to increase its multiplex, adding different beacons with new fluorophores. Impressively, the technology works adequately for individuals testing toward the start of their infection, detecting as little as 0.1 copies/μL in saliva samples with a minimal reaction volume of 10 μL (USD 0.03 per test). The molecular beacon is becoming more attractive over conventional intercalating dyes due to the concern over accidental amplification of nucleic acid targets not of interest [94,95,96,97].

#### 4.1.3. One-Pot Fluorescence Detection

Both intercalating dyes and FRET-based fluorescent probes are adaptable to one-pot set-ups. One-pot fluorescence-based assays keep all reagents in one confined space, minimize human-based error by decreasing the steps to result, and lower the cost when compared to assays requiring multiple substrates. A variety of previously published amplification techniques have been paired with one-pot assays for multiplex or parallel fluorescent detection of SARS-CoV-2 targets of interest [98,99,100]. Notably, a team led by Bhadra et al. developed a one-pot LAMP assay that can be qualitatively analyzed via oligonucleotide strand exchange (OSD) probes that are sequence specific [101]. These probes are partial DNA duplexes that undergo a toehold-mediated strand displacement reaction that separates the longer strand with the fluorophore, which is hybridized to nucleic acids of interest, from the shorter strand with the quencher molecule, which is eventually released. This LAMP-OSD assay is sensitive to as little as 10 copies per reaction and is translatable to at-home testing kits. For the simplest and most cost-effective way to neutralize the amplification inhibitors, simple equipment, such as a water bath or heat block, can be used [64].

Although one-pot reactions are popular in LAMP-based assays, PCR-based assays also commonly utilize a one-pot set-up. Yan et al. proposed a system to reduce false negative and false positive rates by fabricating and developing a co-detection platform for nucleic acid and protein biomarkers in a complex, one-pot sample [102]. Their system, shown in Figure 4f, is a one-pot pre-coated interface proximity extension (OPIPE) assay. It uses RT-PCR techniques to amplify RdRp and E genes from pre-coated tubes with RNA fragments of interest (forming cDNA). From there, TaqMan probes with different fluorophores are added to amplify the target molecules. This assay demonstrates low cross-reactivity at a high sensitivity (10 viral RNA copies/μL) [102]. 

Lastly, Ding et al.’s multiplexed LAMP-based assay for one-pot fluorescent detection of SARS-CoV-2 uses “Proofman”, a proofreading enzyme-mediated probe (the mechanism is highlighted in Figure 4g). When the Proofman probe recognizes a deliberatively placed single nucleotide mismatch on the 3′ end of the probe complementary to a particular SARS-CoV-2 target of interest, a fluorescent signal can be generated and analyzed under a transilluminator [103]. The Proofman enzyme is resemblant of DNA polymerase, checking that the nucleic acids on each phosphate backbone are complementary. The SNV mismatch triggers a cleavage of the primer by the proofreading enzyme, releasing a fluorescent probe from the grasp of a nearby quencher molecule. Coined as the “Duplex RT-Proofman-LAMP” assay, signals representing either the N or ORF1ab genes show up on the FAM or HEX channels, respectively, after 50 min [103].

### 4.2. Colorimetry

Colorimetric readout is a common method for many recently developed SARS-CoV-2 tests due to its simplicity, accessibility, and affordability. Benchtop colorimeters measuring visual light absorbance and plate readers are common in lab settings, but colorimetric measurements can be designed using simple equipment. Colorimetry is particularly attractive in developing POCT for untrained users; for example, many at-home rapid tests utilize paper-based LFAs with a colorimetric readout. Colorimetric readout is most often achieved using a pH indicator [64,104,105] or nanoparticle aggregation [105,106,107]. Assays leveraging colorimetric readout typically include multiple separate reactions performed in parallel, enabling de facto multiplex detection. 

During amplification, a pH decrease is caused by the production of protons as more nucleic acids are produced. Within a weakly buffered solution, pH-sensitive dyes, such as phenol red, will change color as the pH shifts during amplification [105]. Acid-base color readouts are time sensitive and do not exhibit a color change indefinitely, which may be caused by nucleic acid degradation [108]; the color change must be observed within 30 to 60 min to remain sensitive, which could pose a restriction. Nevertheless, this technique provides a straightforward way to analyze readouts, typically observed in a 1.5 mL tube, providing a screening method for further readout development, such as fluorescence [108] or electrochemical [109]. 

**Figure 5:**
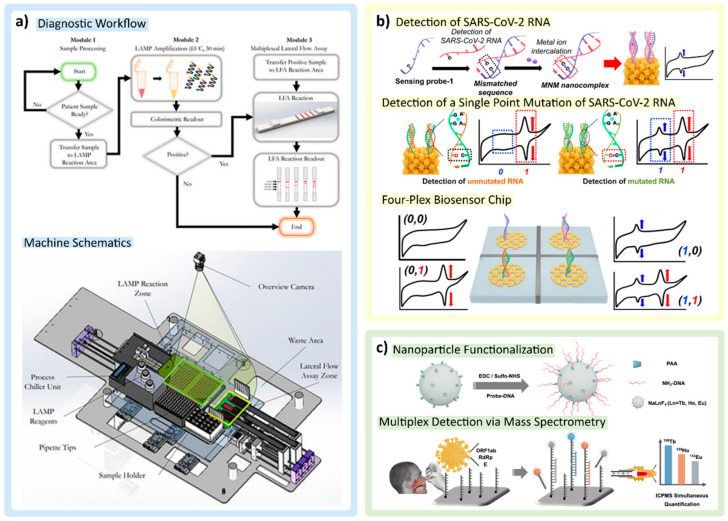
Colorimetric and electrochemical detection techniques: (**a**) Colorimetric detection methods using pH indicator in LAMP reagent mix and gold nanoparticle aggregation on the paper strips are combined into one workflow and implemented within a workstation machine for high-throughput detection. Adapted with permission from Ref. [105]. Copyright 2022, the Authors. (**b**) Electrochemical detection of redox signal from mismatched nucleic acid–metal ion nanocomplex. Adapted with permission from Ref. [88]. Copyright 2022, American Chemical Society. (**c**) Lanthanide nanoparticle (LnNP)-tagging strategy coupled with mass spectrometry for multiplex detection. Adapted with permission from Ref. [83]. Copyright 2021, American Chemical Society.

In an interesting example, Waller et al. developed LAMP assays that use both pH-based and LFA-based colorimetric readouts in a single workflow for high-traffic testing of SARS-CoV-2 (Figure 5a) [105]. The LAMP assay can be achieved in a 96-well plate for high throughput, and the binary readout can be analyzed quickly by a camera in an automated process before further testing the positive wells for specific genes on the LFA [105]. Using logical negative controls, multiplex detection can be implemented with only two colors in a separate reaction. Compared to creating distinct detection/readout sections on paper, this binary readout logic can be expanded to more targets. To multiplex LFA, target sequences are tagged using different fluorescein molecules, and their specific antibodies are striped on the paper-based chip. In the presence of target amplicons, fluorescein is attracted by its antibody pulling target amplicons to the striped area. These amplicons contain biotin on the other end, which attracts streptavidin gold nanoparticles, resulting in red color as they aggregate.

Nanoparticles offer another common colorimetric readout. Colorimetry with nanoparticles is dependent on particle diameter and the mean distance of nanoparticles to the other neighboring particles. Nanoparticles are also liable to aggregate, shrinking the mean distance between each entity, ultimately changing the color that can be visualized on the solution/substrate they reside in/on. Particle aggregation also produces a detectable electrochemical change, as well as a change in fluorescent intensity [110,111]. López-Valls et al. explore several methods of utilizing colorimetric gold nanoparticles (AuNPs) aggregation in conjunction with Cas enzymes. AuNPs can be conjugated with ssDNA oligos and bound together using ssDNA linkers to physically entangle AuNPs. Cas12a complexes, activated when bound to the target gene (amplified by PCR or RPA), cleave the ssDNA linker binding the AuNPs. Following centrifugation, positive solutions will have dispersed AuNPs, resulting in a color change [81]. They also develop a Cas13–crRNA complex, which, when activated by a target sequence, triggers gold nanoparticle aggregation, creating a pink to purple color shift due to the spatial distribution of the AuNPs [81]. This optical readout can be difficult to distinguish with a naked eye in suspension, requiring micro-centrifugation for more distinct readouts.

### 4.3. Electrochemistry

Electrochemical signal is also a powerful alternative readout utilized for developing rapid and sensitive SARS-CoV-2 diagnostic tests. This signal is derived by transducing the biological event into an electrical signal, typically with electrodes employed for biomolecule immobilization and electron movement [112,113]. Electrochemical biosensors have been explored for multiplex detection of SARS-CoV-2 RNA by many research groups, being incorporated into different NAATs [73,114], CRISPR-based techniques [73], and amplification-free tests [80,86,88], similar to optical readout assays. However, electrochemical biosensors do not require optical instrumentation. Instead, the voltage, current, or impedance signals can be derived in correspondence to target analytes using appropriate circuitry for detection.

Chaibun et al. developed ultrasensitive multiplex electrochemical biosensors that simultaneously detect two genes (N and S genes) using isothermal rolling circle amplification (RCA) with padlock ligation and a one-step sandwich hybridization assay. To target two different genes in a single reaction, specific circular DNA templates are designed for RCA, and two redox-active labels are coated on the silica beads together with reporter probes (Si-RP) corresponding to target RCA amplicons. Si-RPs and targets are mixed with the magnetic beads coated with the capture probes (CP-MNB), forming a sandwich hybridization where the target is hybridized by both Si-RP and CP-MNB. The hybridization of two redox labels generates distinct current signals that can be detected by differential pulse voltammetry (DPV) [114]. 

Many research groups have shown that electrochemical assays have the potential to detect nucleic acid targets without any amplification or in complex clinical samples without purification, regardless of the amplification techniques available to improve sensitivity [80,86,88]. Li et al. demonstrated an ultrasensitive biosensing technique utilizing the trans-cleavage mechanism of CRISPR Cas13a and the graphene field-effect transistor (gFET). gFET is a three-terminal electronic device in which the external electric field can be applied through the gate electrode to control the source–drain current passing through a single carbon atom thick graphene semiconductor channel. The mechanism of CRISPR Cas13a-gFET relies on the positive shift of charge neutrality point voltage of the gFET, which is initially functionalized with a negatively charged RNA reporter immobilized on the graphene surface. If the targets are present, the activated Cas13a endonucleases cleave negatively charged reporters off the gFET surface, resulting in reducing the electron transfer to the graphene channel. To allow multiple parallel detections of targets of interest with a single sample input and avoid variations from sensor to sensor, the device consists of an array of six CRISPR Cas13a-gFETs on a silicon wafer. The detection of the SARS-CoV-2 N gene against respiratory syncytial virus (RSV) genome was demonstrated with a LOD down to 0.6 copies/μL [80].

Another multiplexed nucleic acid amplification-free electrochemical biosensor is reported by Kashefi-Kheyrabadi et al. using four-way junction (4-WJ) hybridization. The structure of 4-WJ is formed by a universal DNA hairpin (UDH) probe hybridized by two adaptor strands and a SARS-CoV-2 RNA target. To generate the signal, one of the adaptor strands is functionalized with a redox marker. This device can simultaneously detect S and ORF1ab genes by using different redox markers. The UDH probe is immobilized to the gold nanoneedle structured electrode. This assay can be performed in a single step by incubating all the biosensor components together. In the presence of targets, the adaptor strands hybridize to both the RNA target and UDH that switches from the hairpin structure to a straight strand, forming 4-WJ and causing the redox markers to approach the electrode surface. This phenomenon facilitates the electron transfer between redox probes and the electrode surface, generating electrochemical signals, which can be detected by DPV [86].

One of the largest difficulties in nucleic acid detection for SARS-CoV-2 is the frequency of mutations that the virus undergoes. Interestingly, Yoon et al. use this phenomenon to their advantage by allowing metal ions to form a mismatched nucleic acid–metal ion (MNM) nanocomplex shown in Figure 5b. Metal ions are advantageous, as they provide excellent redox signal for electrochemical readout, and in instances where there are two or more mutations in SARS-CoV-2 RNA, mutations can be distinguished at the single nucleotide level using various ionic probes (e.g., Mg, Au). The addition of multiple intercalating metal probes allows for multiplex detection, distinguishing SARS-CoV-2 wild-type infections compared to mutant. Moreover, Yoon et al. developed the Nanoporous Electrode Array (NPEA) that could enhance the redox signal further due to its ability to provide enhanced electron transfer. Sensing probes can be scattered across the NPEA, hybridizing with SARS-CoV-2 RNA of the complementary sequence, immobilizing the nucleic acid sequences of significance, and allowing the metal ions to intercalate [88].

### 4.4. Other Readout Modality 

Li et al. reported an intriguing multiplex detection method by using a lanthanide nanoparticle (LnNP)-tagging strategy and leveraging mass spectrometry as the readout modality (Figure 5c). In this method, three LnNPs (Tb, Ho, and Eu alloys) were designed to target the ORF1ab, RdRp, and E genes of SARS-CoV-2 RNA. LnNPs, coated with polyacrylic acid, provide excess carboxylic acid groups for further bioconjugation with specific amine-functionalized capture DNAs. The magnetic bead (MB) was also conjugated with three variations of capture DNAs. In the presence of target genes, the target RNA hybridizes and anchors to the corresponding DNA probe on the MB, and the half-remaining chain could also hybridize with the corresponding LnNP-based nanoprobes as a sandwich-like structure. By applying the enhancer solution, LnNP tags were digested and released three different Ln ions corresponding to different targets that could be detected and distinguished by ultrasensitive inductively coupled plasma mass spectrometry (ICPMS) detection. The sample consumption and material costs of this test are low; combined with the short analysis time of ICPMS, LnNP tagging is an attractive protocol for detecting strand types with high accuracy [83].

## 5. Point-of-Care Amenable Platforms

**Figure 6:**
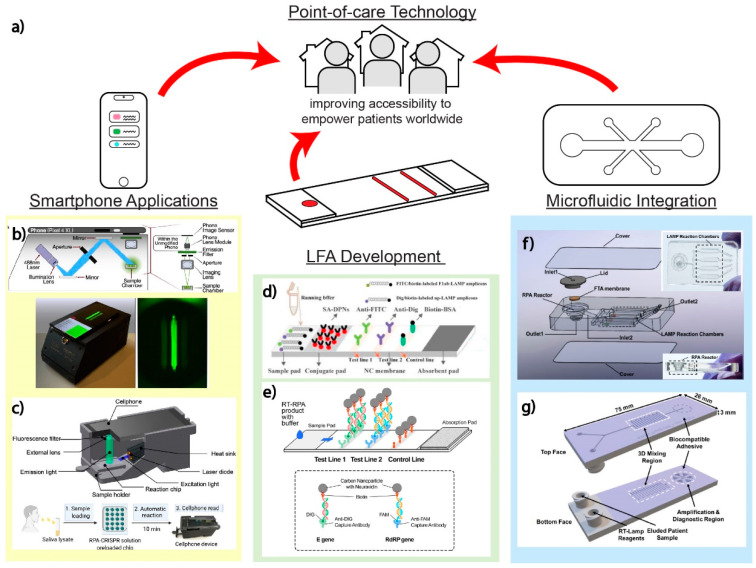
Point-of-care technologies. (**a**) POCT for improving diagnostic testing accessibility to medical deserts, especially in lower income communities around the world. The use of smartphone technology, microfluidic integration, and/or continued LFA development is pertinent to empowering individuals without high-resource clinicians and hospitals nearby. (**b**) Smartphone-based detection for amplification-free detection of SARS-CoV-2 with CRISPR-Cas13a. Adapted with permission from Ref. [12]. Copyright 2021, Elsevier. (**c**) Smartphone-based detection of VOCs by PAM-targeting mutations. Adapted with permission from Ref. [118]. (**d**) Lateral flow assay using nanoparticles. Adapted with permission from Ref. [107]. Copyright 2020, Elsevier. (**e**) Lateral flow assay with RPA for low resource setting. Adapted with permission from Ref. [69]. Copyright 2021, Elsevier. (**f**) 3D-printed integrated microfluidic chip for colorimetric detection. Adapted with permission from Ref. [117]. Copyright 2021, Elsevier. (**g**) Microfluidic chip with LAMP-based portable assay. Adapted with permission from Ref. [121]. Copyright 2020, the Authors.

Ideally, multiplexed diagnostic testing for SARS-CoV-2, SARS-CoV-2 variants, and other respiratory viruses should still be amenable for POC use. To this end, smartphone technology, LFAs, and microfluidics offer potential solutions that promise improved accessibility for all patients worldwide, empowering them to diagnose themselves without in-person medical personnel and equipment (Figure 6a). We highlight in this section the promising assays and platforms for detecting SARS-CoV-2, SARS-CoV-2 variants, and other respiratory viruses at POC. 

### 5.1. Smartphone Detection

The smartphone has in recent years become a promising tool for developing accessible diagnostic devices, since it can serve as a simple reader due to a fully integrated sensor, data processor, as well as a user interface in a pocket-size package [115]. Many smartphone applications utilize paper-based assays and NP enhancement techniques with a simple colorimetric readout, from which, commonly, with the naked eye, only binary signals (yes/no) can be determined. However, complementary metal oxide semiconductor (CMOS) cameras within smartphones are now capable of customized control or automatically optimizing white balance, color balance, and autofocus, which enable differentiation between the ranges of colors and intensities outside the human visual spectrum limitations, allowing the inspection of both fluorescence and colorimetric readouts [115,116]. Smartphones integrated with fabricated handheld devices provide improvement for the POCT, as they are continuously being modernized and revamped to meet new challenges. Multiple assay-specific apps have been created, which increase the ease of use for smartphone-based platforms [66,69]. Advances in smartphone capabilities allow many laboratory methods readily adaptable to POCT applications, which are very useful, especially in a pandemic situation, such as the COVID-19 pandemic, where the accessibility, cost, and availability are important factors to cope with the situation. 

Thus, unsurprisingly, there have been several smartphone-based multiplex SARS-CoV-2 diagnostic assays in the past few years [9,12,66,77,117]. Fozouni et al. built a mobile phone fluorescent microscope with a 488 nm LED and filter to detect the green fluorescence spectra shown in Figure 6b. Images can be captured by the mobile phone, with offline analysis using a MATLAB code, but it still requires manual inputs for the region of interest [12]. Similarly, Ning et al. designed a smartphone-based fluorescent microscope, which is equipped with a 465 nm laser diode for excitation and a 500 nm emission filter, for capturing images, shown in Figure 6c [118]. The same smartphone-based fluorescent microscope was later used by the same research team to detect multiple SARS-CoV-2 variants [9]. Using a smartphone in combination with image processing, the real-time readout and accuracy can be improved, as demonstrated by Nguyen et al. targeting multiple SARS-CoV-2 genes [66]. Song et al. captured fluorescent images of the reactions inside a dark box with a smartphone, paired with a web-based Python application and machine learning to fully automate ROI selection and provide nM specific target detection [77]. Another example achieved by Yin et al. demonstrated the use of an app that was developed to distinguish even slight changes in wastewater samples using colorimetric readout. The app allows wastewater testing in remote areas with instant data transfer, allowing real-time data tracking [117]. 

### 5.2. Lateral Flow Assays

LFAs are popular platforms for POCT due to their flexibility, low cost, and ease of fabrication for mass-scale production, which could be very beneficial when dealing with a situation such as the COVID-19 pandemic, e.g., when simplicity and a large number of tests are needed. LFAs are typically performed by loading a sample onto the paper chip, and the capillary action of the paper allows the sample to flow and mix with the pre-loaded reagents, inducing a chemical reaction that enables colorimetric or fluorescence readouts. Some researchers utilized CRISPR/Cas systems [20,70,78] or OSD probes [64] combined with LFA to enhance specificity [20,64]. For example, Arizti-Sanz et al. simplified a CRISPR/Cas13-based assay for SARS-CoV-2 VOCs identification using colorimetric lateral-flow strip and lyophilized reagents [20]. Additionally, as mentioned previously, Bhadra et al. demonstrated multiplexed LAMP-OSD tested with human saliva spiked with SARS-CoV-2 virions utilizing Boolean and logic-gated colorimetric readout on lateral flow dipsticks [64].

Other techniques that allow for the visualization of paper-based readout for multiplex detection are utilizing nanoparticles or microparticles aggregation and immunoassay application. LFAs often use strong biotin and streptavidin binding interaction for detection mechanisms that can be applied with appropriate labeled antigens/antibodies and AuNPs. As shown in Figure 6d, dye-coated, streptavidin-conjugated polymeric nanoparticles flow through and bind to the biotin; through the NP aggregations, visible multiplexed detections can be read [105,107]. In another example utilizing nucleic acid amplification, RPA primers labeled with biotin on the 5′ end and digoxigenin (hapten) on the 3′ can also bind with neutravidin-conjugated carbon NPs on specific regions on a lateral flow test demonstrated by Cherkaoui et al., highlighted in Figure 6e [69]. Additionally, Wang et al. developed an amplification-free nucleic acid immunoassay, implemented on a lateral flow strip for detecting SARS-CoV-2 genes by using DNA probe functionalized fluorescent nanoparticles labeled with an antibody that binds to double-stranded DNA–RNA hybrids for signal amplification [119].

### 5.3. Microfluidic Integrated Platforms

Although PCR amplification may require bulky and expensive lab equipment to perform the temperature fluctuation in the expansion process, as well as intense, trained human labor, Trick et al. were able to overcome these limitations by integrating a magnetofluidic cartridge into an automated and portable PCR testing platform [120]. This tool utilized inexpensive components (e.g., 3D printing, laser cutting, and programed microcontroller) that combined the sample preparation steps and PCR to provide results in less than 30 min with minimal training required. The multiplexing is achieved by two fluorescence probes to identify B.1.1.7/B.1.351 variants and SARS-CoV-2/Influenza with a LOD of 12.5 copies/µL [120].

Yin et al. developed a 3D-printed integrated microfluidic chip capable of colorimetric detection of SARS-CoV-2 and other pathogens in wastewater, as shown in Figure 6f. The microfluidic chip, fabricated from a clear methacrylate-based resin, supports two individual reactor units: an RPA reactor unit enabling nucleic acid extraction and a SEC-LAMP reactor array [117]. Uniquely, the system multiplexes detection of both SARS-CoV-2 and human enteric pathogens. With a sensitivity of 100 GE/mL and 500 CFU/mL for SARS-CoV-2 and human enteric pathogens, this technology enables portable, on-site, inexpensive (~USD 2.00 per assay) testing of disease outbreaks in broader community-wide populations [117].

Additionally, another LAMP-based portable assay developed by Ganguli et al. is a 3D cartridge with an inlet region, mixing region, and amplification and diagnostic region (Figure 6g). This allows for the mixing of the patient sample and RT-LAMP reagents into the amplification chamber on the microfluidic device, and a smartphone can be used for a simple detection system. The Luer locks on the chip enable simple sample loading from syringes before undergoing mixing in a serpentine pattern downstream [121]. The cartridge is fully disposable after being printed with rigid polyurethane on a Carbon M2 printer, and a biocompatible adhesive seals the top and the bottom of the device together. This cartridge is used in a platform, which can display results in under 40 min and has a LOD of 50 copies/μL [121].

Ding et al.’s development of a resin 3D-printed lab-on-disc platform with a similar multiplex and run time (three multiplexes, 50 min) has a one-step process for users. Instead of strict RT-LAMP, the group utilizes centrifugation, enabling the assay to remove residual volume, unused samples or reagents, or any bubbles in the system that could interfere with diagnosis. Although in theory, the centrifugation should enhance the specificity of the assay, it was 90% specific [122]. 

## 6. Remaining Challenges and Future Perspectives

Multiplex nucleic acid diagnostic testing has advanced rapidly in the past two years (Table 1). Despite considerable advances, significant challenges remain for developing multiplex diagnostic tests for combating COVID-19. We discuss in this section the key remaining challenges that must be addressed to propel further advances. In addition, we share our perspectives on future trends for research and development.

### 6.1. Remaining Challenges

All methods and platforms reviewed herein have their strengths but also shortcomings that should be addressed to further advance multiplex nucleic acid diagnostic testing for combating COVID-19. First, the various current reaction techniques reviewed herein have their unique remaining challenges. Despite reports of rapid and miniaturized RT-PCR [18,120], RT-PCR often still involves lengthy assay time and relies on a bulky thermocycler. RT-LAMP can still be susceptible to false amplification [56,124]. Although the combination of RT-RPA and CRISPR has garnered significant research interests, RT-RPA is infrequently deployed alone, while most existing CRISPR assays are multi-step assays that incorporate pre-amplification, which increases assay complexity and assay time [58,125], whereas single-step amplification-free CRISPR assay [12] must still improve in sensitivity. Other reaction techniques reviewed herein are nascent, so their usefulness is best assessed later. Second, the detection modalities reviewed herein have a common remaining challenge, in that they are restricted to low-plex detection. Indeed, fluorescent detection is limited by the spectral overlap of fluorophores [59,126], colorimetric detection is essentially binary, and electrochemical detection is limited by the number of electrodes. Finally, the point-of-care amenable platforms reviewed herein also have their unique remaining challenges. Smartphone-based platforms face hurdles in standardization due to lack of standardization across smartphone operating systems. LFAs are simple but insensitive. Microfluidic platforms, despite significant research efforts, are currently still complex for untrained users and mostly laboratory bound.

A general remaining challenge is the development of high-plex platforms that can keep pace with genetic mutations required for tracking SARS-CoV-2 variants but are simultaneously amenable for POC use. For example, although the powerful mCARMEN platform [71] can detect 18 SARS-CoV-2 mutations and 21 viruses, it still requires separate sample preparation, a commercial microfluidic device, and a bulky instrument, which significantly hampers its potential for POC use. On the other hand, the current POC-amenable platforms predominantly only detect fewer than four targets. Such four-plex detection would be useful for identifying SARS-CoV-2 from other circulating respiratory viruses. However, as SARS-CoV-2 continues to mutate, and variants inevitably emerge, the development of platforms that can achieve high-plex detection while maintaining POC amenability could play an important role.

Finally, the most notable remaining challenge is the clinical deployment of multiplexed nucleic acid diagnostic testing—a challenge that extends beyond integrating the functional reaction technique with the detection modality within a POC-amenable platform. Clinical use requires the consideration of specimen type and collection method, incorporation of sample preparation, and further implementation of a user-friendly platform with simple user interface, easy-to-interpret readout, and minimal user intervention in a sample-to-result workflow [124]. For SARS-CoV-2, sample preparation from predominant clinical samples, including nasopharyngeal swabs and saliva, has become straightforward [127,128], but most of the research to date has yet to focus on incorporating sample preparation or implementing platforms. Furthermore, new multiplexed nucleic acid diagnostic assays and platforms must be validated with more rigorous patient-collected sample testing to determine whether they can indeed avoid false positives from other pathogens. Assay cost represents a critical factor for the clinical uptake of diagnostic testing. Most multiplexed nucleic-acid-based viral diagnostic testing research reported to date, however, emphasizes feasibility demonstration. The cost of these methods remains to be fully evaluated. Finally, manufacturing and reagent storage represent additional challenges. It is important to consider the rapid mass production of inexpensive tests adequate for multiplexing various SARS-CoV-2 variants of the present and the future to save on manufacturing costs. Likewise, it is important to consider reagent storage, such that the diagnostic tests can be transported and deployed even without the cold chain. Despite intense research and development to date, there are still plenty of remaining challenges (and opportunities).

### 6.2. Future Perspectives

Ideally, the advances in POC-amenable multiplexed diagnostic testing should continue. In addition to the more traditional scenarios for POC testing, such as local clinics and testing sites, we see two additional scenarios—self-testing and wastewater monitoring—as potential future trends. Self-testing could ideally allow individuals with suspected SARS-CoV-2 infections to determine their infection status without a trip to local clinics and testing sites, which could increase the risk of transmission of SARS-CoV-2 and perhaps other pathogens. Toward developing such self-testing devices, we find the device recently reported by Tang et al. promising. This mobile device works in tandem with a cartridge that houses a lysis chamber and three RT-LAMP chambers, such that it can directly detect SARS-CoV-2 from the saliva of an individual with suspected infection [129]. Although this device currently performs only single-plex detection, the multiple reaction chambers within the cartridge suggest multiplexed detection can be achieved. Additionally, it is foreseeable that self-testing may also simultaneously target SARS-CoV-2 RNA and anti-SARS-CoV-2 antibodies, as demonstrated by Najjar et al. in a microfluidic device [130] and by Masterson et al. via their nanoplasmonic biosensors [131]. Wastewater monitoring is an emerging strategy for tracking or even anticipating the dynamics of SARS-CoV-2 infection at the community scale [132,133]. The timeliness of this strategy may be enhanced through portable diagnostic platforms that can be deployed on site. To this end, the POC device reported by Trick et al. [120] may present a potential solution, although the sample preparation from a large volume of wastewater must be tailored and could be challenging. For both self-testing and wastewater monitoring, Bluetooth and smartphone technology could enable timely detection with geographic location and potentially reveal demographic trends in a private and secure manner.

For a broad development of multiplexed diagnostic tests in the future, regardless of the reaction techniques and detection modalities, it is perhaps prudent to embrace the approach of simultaneously parallelizing multiple single-plex assays in separate chambers located in a single device instead of developing true multiplex assays in one pot. This parallelized approach takes advantage of the modularity of a single-plex assay, where a single-plex assay designed to detect a target that has become outdated (e.g., a mutation associated with an outdated variant) can be readily replaced by a single-plex assay detecting an up-to-date target without disrupting other single-plex assays from detecting their respective targets. The disadvantage of this approach would arise from the increased number of reactions, which increases reagent and material consumption, and hence, assay cost. Microfluidics presents a potential tool for reducing reagent consumption and cost, though at the expense of additional material and instrumentation cost.

Although in this review we emphasized POC applications, we believe multiplexed nucleic acid testing could still be useful within a laboratory setting. For example, multiplexed nucleic acid testing could be implemented within a hypothetical tiered workflow to bridge the gap between POC tests that focus on identifying SARS-CoV-2 cases and sequencing that focuses on discovering new genetic mutations and SARS-CoV-2 variants. In this workflow, the multiplexed nucleic acid testing assay can be designed to target a panel of genetic mutations, such that the mutation pattern of each sample can be used to confirm the initial positive diagnosis and to provide a screening mechanism for sequencing. As only suspected SARS-CoV-2 positive samples are to be analyzed via such a multiplexed nucleic acid testing assay, the demand for testing throughput is alleviated. Moreover, as samples with unfamiliar mutation patterns can be selected for sequencing, the sequencing-based discovery of new mutations and variants can be more targeted. Designing such multiplexed nucleic acid testing assays could be challenging, especially as SARS-CoV-2 variants continue to emerge. To stay ahead of the emergence of SARS-CoV-2 variants, it may be useful to target the mutation “hotspots” predicted via computation [134] or in vitro evolution [135] methods.

## 7. Conclusions

Multiplex nucleic acid diagnostic testing has advanced rapidly in response to the SARS-CoV-2 pandemic and has shown strong potential for minimizing false negatives, detecting specific VOCs, and improving clinical accessibility. Herein, we reviewed notable advances in multiplex nucleic acid diagnostic testing, including reaction techniques, detection methods, and POC-amenable platforms. Among the reaction techniques, RT-PCR remains the gold standard, but isothermal techniques—most notably RT-LAMP and CRISPR—have surged in popularity. Similarly, fluorescence-based detection remains the go-to detection modality, although colorimetric detection and electrochemical detection have attracted some attention. Among the POC-amenable platforms, smartphone-based platforms have been used to detect both fluorescence and colorimetric diagnostic readouts and continue their upward trend. Despite these advances, there are remaining challenges—or research opportunities—in addressing the shortcomings of all existing reaction techniques, detection modalities, and POC-amenable platforms, as well as developing true POC and high-plex diagnostic tests that can keep pace with SARS-CoV-2. Furthermore, most innovations reviewed herein must still be rigorously validated and further advanced, especially in platform integration, manufacturing, cost, and reagent storage. Nevertheless, multiplex nucleic acid detection of viral diseases is a research vein bursting with potential to impact both individuals and public health at large. The technologies reviewed here will continue to broaden the capabilities of rapid, highly specific disease detection and point-of-care testing as a field.

## Figures and Tables

**Table 1:** Summary table of multiplex nucleic acid diagnostic tests for SARS-CoV-2.

Features	Target	Amp	Detection	Sample	Test Time	LOD (cps/µL)	Clinical Sensitivity	Clinical Specificity
PAM-targeting CRISPR/Cas12a [9]	VOC	RPA	Fl ^1^	Nas	1 h	0.5	61/70	12/21
Plasmonic NP for POCT [18]	Ge	qPCR	Fl	Sal	0.4 h	6	10/10	9/9
Molecular beacon POCT [19]	Ge	LAMP	Dual Fl ^1^	Sal	1.3 h	100	16/17	24/24
Body-heat-based Amp + Cas13 [20]	VOC	RPA	C ^2^	Nas	1.5 h	100	38/42	30/30
Centrifugal microfluidic disc [42]	RV	direct dPCR	Fl ^3^	Nas	1.5 h	2	548/555	3020/3034
ARMS-PCR + quantum dot detection [47]	VOC	ARMS PCR	Dual Fl ^1,2^	Sal (Syn)	2 h	500	--	--
Mutation detection in large populations [56]	VOC	dPCR	Fl	WW	2 h	3	--	--
False positive detection (OSD) probes [64]	Ge	LAMP	C/Fl ^1,2^	Sal (Syn)	1 h	20	--	--
POCT with smartphone app [66]	RV	LAMP	Fl ^1,3^	Nas	1 h	200	4/4	12/12
Cas12/13 combinatorial detection [71]	VOC	PCR	Fl Barcode	Nas	5 h	500	316/316 127/133 *	167/212 30/33 *
Electrokinetic extraction + Cas12a [73]	Ge	LAMP	Fl ^3^	Nas	0.6 h	10	30/32	32/32
Amp-free Cas13a + reporter [74]	RV	None	Fl	Nas	0.5 h	3.9	5/5	1/1
CRISPR/Cas12a-based technique [75]	RV	RPA	Fl ^3^	Th/Nas	0.5 h	3	68/69	103/103
Machine learning + Cas9 [77]	VOC	LAMP	EChem ^1^	Nas	1 h	100	77/77	59/59
Rolling circle amp silica NP tagging [114]	Ge	RCA	Fl ^3^	Nas	3 h	1	50/50	56/56
CMOS biochip for parallel detection [123]	RV	PCR	Pixel Fl	(Syn)	1.7 h	1	--	--

Target refers to targets that are multiplexed (“Ge” multiple regions from a single strain; “VOC” the same region of multiple variants of concern; “RV” multiple different viral diseases). Amp refers to the nucleic acid amplification technique. Detection refers to the modality used to determine positive/negative result (“FL” fluorescent signal; “C” colorimetric readout; “EChem” electrochemical signal). Sample refers to the collection type the test is designed for (“Nas” nasal pharyngeal; “Sal” saliva collected in a tube; “Th” throat swab; “WW” water collected in a tube. “(Syn)” refers to a synthetically spiked sample). Test Time is calculated from the beginning of the amplification step, including RNA isolation, excluding sample collection, transportation, or preparation). LOD (limit of detection) is the minimum concentration of RNA during detection (listed in copies/µL). With different multiplex targets for each assay, the reported LOD refers to highest individual target LOD. Clinical Sensitivity is reported as the number of true positive cases over the number of true positive cases and false negative cases, and may be summed for multiple tests. Clinical Specificity is reported as the number of true negative cases over the number of true negative cases and false positive cases, and may be summed for multiple tests. Notes: ^1^ smartphone incorporation; ^2^ lateral flow assay; ^3^ microfluidics; * tested in a clinical setting.

## Data Availability

Not applicable.
